# Coordinated increase in inhibitory and excitatory synapses onto retinal ganglion cells during development

**DOI:** 10.1186/1749-8104-6-31

**Published:** 2011-08-24

**Authors:** Florentina Soto, Adam Bleckert, Renate Lewis, Yunhee Kang, Daniel Kerschensteiner, Ann Marie Craig, Rachel OL Wong

**Affiliations:** 1 Department of Biological Structure, University of Washington, 1950 Pacific Ave, Seattle, WA 98195, USA; 2 Department of Ophthalmology and Visual Sciences, Washington University in St Louis, 660 S. Euclid Ave, St Louis, MO 63110, USA; 3 Department of Anatomy and Neurobiology, Washington University in St Louis, 660 S. Euclid Ave, St Louis, MO 63110, USA; 4 Brain Research Centre and Department of Psychiatry, University of British Columbia, 2211 Wesbrook Mall, Vancouver, BC, Canada V6T 2B5

## Abstract

**Background:**

Neuronal output is shaped by a balance of excitation and inhibition. How this balance is attained in the central nervous system during development is not well understood, and is complicated by the fact that, *in vivo*, GABAergic and glycinergic synaptogenesis precedes that of glutamatergic synapses. Here, we determined the distributions of inhibitory postsynaptic sites on the dendritic arbors of individual neurons, and compared their developmental patterns with that of excitatory postsynaptic sites. We focused on retinal ganglion cells (RGCs), the output neurons of the retina, which receive excitatory input from bipolar cells and inhibitory input from amacrine cells. To visualize and map inhibitory postsynaptic sites, we generated transgenic mice in which RGCs express fluorescently tagged Neuroligin 2 (YFP-NL2) under the control of the *Thy1 *promoter. By labeling RGC dendrites biolistically in YFP-NL2-expressing retinas, we were able to map the spatial distribution and thus densities of inhibitory postsynaptic sites on the dendritic arbors of individual large-field RGCs across ages.

**Results:**

We demonstrate that YFP-NL2 is present at inhibitory synapses in the inner plexiform layer by its co-localization with gephyrin, the γ2 subunit of the GABA_A _receptor and glycine receptors. YFP-NL2 puncta were apposed to the vesicular inhibitory transmitter transporter VGAT but not to CtBP2, a marker of presynaptic ribbons found at bipolar cell terminals. Similar patterns of co-localization with synaptic markers were observed for endogenous NL2. We also verified that expression of YFP-NL2 in the transgenic line did not significantly alter spontaneous inhibitory synaptic transmission onto RGCs. Using these mice, we found that, on average, the density of inhibitory synapses on individual arbors increased gradually until eye opening (postnatal day 15). A small centro-peripheral gradient in density found in mature arbors was apparent at the earliest age we examined (postnatal day 8). Unexpectedly, the adult ratio of inhibitory/excitatory postsynaptic sites was rapidly attained, shortly after glutamatergic synaptogenesis commenced (postnatal day 7).

**Conclusion:**

Our observations suggest that bipolar and amacrine cell synaptogenesis onto RGCs appear coordinated to rapidly attain a balanced ratio of excitatory and inhibitory synapse densities prior to the onset of visual experience.

## Background

The normal functioning of the nervous system requires balanced excitatory and inhibitory neurotransmission. If excitation or inhibition is perturbed, neurons undergo alterations in their intrinsic excitability and synaptic transmission in order to restore a balance, and prevent their circuits from undergoing epileptiform activity [1,2]. While such homeostatic plasticity in mature neuronal networks is well studied [3], much less is known concerning how balanced excitation and inhibition is normally achieved during development. In many parts of the central nervous system (CNS), interneurons containing the classical inhibitory transmitters γ-aminobutyric acid (GABA) or glycine form functional synaptic connections well before glutamatergic synapses emerge [4,5]. GABAergic or glycinergic synaptogenesis may thus outpace glutamatergic synaptogenesis, requiring mechanisms to adjust excitation and inhibition to achieve a balance throughout development. In contrast, it is possible that inhibitory and excitatory synapses onto a given neuron develop largely in parallel, maintaining a constant ratio of synapse densities at all stages shortly after glutamatergic synaptogenesis begins. Here, we distinguished between these two possibilities by comparing the densities of inhibitory and excitatory synapses on the dendritic arbors of cells of the same type during the period of synaptogenesis.

Many studies have tracked the distribution of glutamatergic synapses on a given neuron by imaging spines [6-9] or by visualizing fluorescently labeled postsynaptic densities or receptors on the dendrites [10-15]. However, spatial maps of inhibitory synapses across the dendritic arbor of individual neurons during *in vivo *development have not been charted. Because mature inhibitory synapse distributions vary across cell types [16] and even across the arbor of individual neurons [17], it is important to obtain and compare inhibitory and excitatory synapse distributions on the dendrites of the same cell type. Here, we focused on retinal ganglion cells (RGCs) of the vertebrate retina because the physiology and morphology of these neurons are generally well studied. We mapped the spatial distributions of inhibitory postsynaptic sites on mouse RGCs and compared their developmental distributions with those of glutamatergic postsynaptic sites [14].

Inhibition in the inner retina is provided by amacrine interneurons that primarily use either GABA or glycine as their neurotransmitter [18,19]. Serial electron microscopy (EM) of dendritic trees of RGCs in adult macaque, cat and rabbit revealed that amacrine cells contribute a significant fraction of the total number of synapses onto a RGC [20-24]. This fraction, however, differs greatly across species, and even amongst RGC subtypes within a species. For example, in cat, amacrine cells make about 30 to 60% of all synapses onto beta-RGCs [25,26] but about 80 to 86% of synapses onto alpha-RGCs [22,26]. However, the ratio of amacrine to bipolar cell synapses appears to be consistent within a RGC subtype [25]. Combined immunolabeling for ionotropic glutamate receptors and gephyrin recently demonstrated relatively similar densities of excitatory and inhibitory synapses on individually labeled small bistratified [27] and sparse or thorny arbored [28] RGCs of the primate retina.

The ratio of amacrine to bipolar cell synapses is not known for developing RGCs. EM of the mouse retina suggested that conventional (presumed amacrine) synapses are present shortly after birth, and ribbon (bipolar) synapses appear only after postnatal day (P)10 [29]. Conventional synapse density increases rapidly as ribbon synapses form. This increase in conventional synapses may represent increased amacrine synaptogenesis onto RGC dendrites and/or synaptogenesis largely onto the newly formed axonal terminals of differentiating bipolar cells [30]. However, it is also possible that early in development, conventional synapses include bipolar synapses that have not yet localized ribbons to their presynaptic sites [31,32]. Thus, in order to distinguish amacrine and bipolar cell synapses onto developing RGCs, excitatory and inhibitory postsynaptic markers are necessary.

We previously expressed PSD95 fluorescently tagged with yellow fluorescent protein (PSD95-YFP) in RGCs to label their glutamatergic postsynaptic densities [14]. Here, we visualized inhibitory postsynaptic sites on RGCs in transgenic mice in which Neuroligin 2 (NL2) was tagged with YFP (YFP-NL2). Neuroligins are postsynaptic cell adhesion molecules that interact with presynaptic neurexins and are essential for normal synaptic maturation and function [33]. NL2 is selectively expressed at inhibitory synapses in the CNS [34,35]. Because NL2 protein is present at early stages of differentiating inhibitory synapses [35,36], we used YFP-NL2-expressing transgenic mice as a means of identifying inhibitory postsynaptic sites on the RGCs. We labeled the dendrites of large-field RGCs and mapped the spatial distribution of YFP-NL2 puncta on their dendritic arbors across different ages. We found that the density of inhibitory postsynaptic sites on large-field RGCs increases gradually with maturation, following a time course similar to that for their glutamatergic postsynaptic sites. Interestingly, the ratio of YFP-NL2 and PSD95-YFP puncta density per cell remained constant shortly after bipolar cell synapses form, suggesting that excitatory and inhibitory synaptogenesis onto RGCs may be coordinated, perhaps to ensure that neuronal excitability is suitably regulated and stable throughout development.

## Results

### Neuroligin 2 localizes to inhibitory postsynaptic sites in the retina

**Figure 1:**
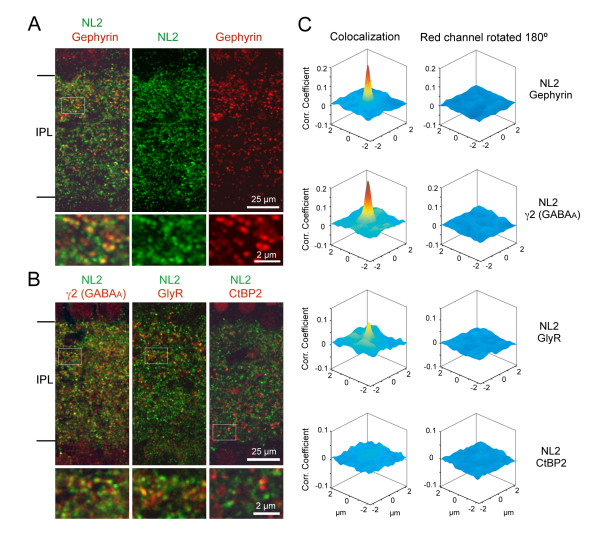
**NL2 colocalizes with markers of inhibitory synapses in wild-type (WT) mice**. **(A, B) **Single plane confocal images of P21 to P25 vertical sections of WT retina labeled with antibodies against NL2 and the inhibitory or excitatory synapse markers gephyrin, γ2 subunit of GABA_A _receptors or glycine receptors (GlyR) and ribbons (carboxy-terminal binding protein 2 (CtBP2)). IPL, inner plexiform layer. Higher magnification views (smaller panels) are shown for the boxed regions in (A, B). **(C) **Two-dimensional cross-correlation coefficients of pixel intensities in the red and green channels are plotted to demonstrate whether the signals show non-random colocalization (peak at 0,0), or random colocalization (flat distribution). See Materials and methods for analysis. As a control for random colocalization of signals, correlation plots were obtained after the red channel was rotated 180° relative to the green (NL2) channel.

In order to compare the distribution of fluorescently tagged NL2 in our transgenic mouse lines with the endogenous expression of NL2, we first carried out immunostaining of endogenous NL2 and inhibitory postsynaptic markers in wild-type retina. In the rodent retina, gephyrin appears to be present mostly at glycinergic synapses and at a subset of GABAergic synapses mainly containing the α2 subunit of the GABA_A _receptor [37]. Figure 1A shows immunolabeling for endogenous NL2 and gephryin in the inner plexiform layer (IPL) of a P21 retina, and illustrates the co-localization of these two postsynaptic proteins. To ascertain whether NL2 is present at both glycinergic and GABAergic synapses in the IPL, we combined immunolabeling for NL2 with immunostaining using an antibody against the γ2 subunit of the GABA_A _receptor (gift of JM Fristchy [38]), or an antibody that recognizes all subunits of the glycine receptor (mAb4a) [39]. Similar to previous observations [40], we found that in the IPL, NL2 colocalized with the γ2 subunit of the GABA_A _receptor, and with glycine receptors to some extent (Figure 1B).

We performed two-dimensional cross-correlation analysis to determine whether colocalization between two labels was real or random (see Materials and methods). Our results indicate that there is a positive correlation of endogenous NL2 signal and immunolabeled gephyrin, GABA_A _and glycine receptors (Figure 1C). We confirmed that such correlations were not an artifact of the analysis by repeating the calculation with one channel rotated by 180°. Hoon *et al*. [40] demonstrated that endogenous NL2 does not colocalize with the scaffolding protein PSD95, found at excitatory postsynaptic sites in the retinal IPL. We similarly found that carboxy-terminal binding protein 2 (CtBP2), a marker of bipolar cell ribbons at glutamatergic presynaptic release sites, was not apposed to endogenous NL2 clusters (Figure 1B, C). Taken together, our observations concur with past observations [40] indicating that NL2 is a suitable marker of inhibitory postsynaptic sites in the retinal IPL.

### YFP-NL2 expression in the transgenic retina matches the distribution of the endogenous protein

**Figure 2:**
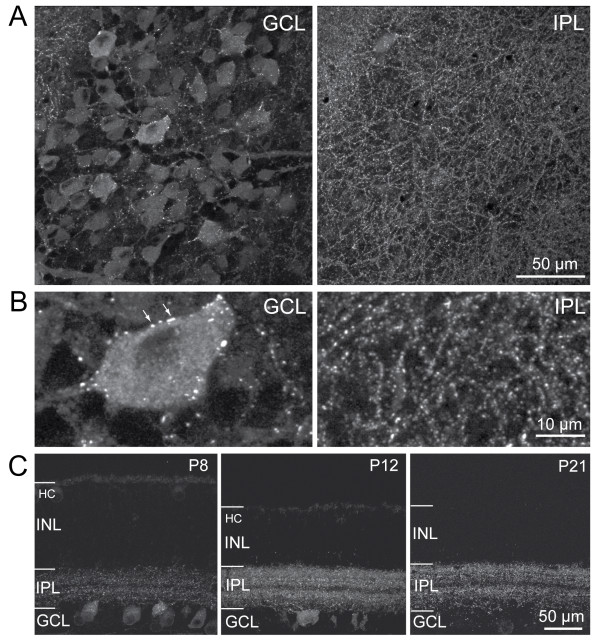
**Punctate expression is observed in the cell bodies in the ganglion cell layer (GCL) and in the inner plexiform layer (IPL) of *Thy1-YFP-NL2 *retinas**. **(A) ***En face *(top) view of a P21 *Thy1-YFP-NL2 *retina at the level of the RGC layer or at the IPL. **(B) **High magnification view of YFP-NL2 puncta in the GCL and IPL. Arrows indicate puncta on a RGC soma. (A, B) Maximal intensity projection encompassing 4.5 μm thickness. **(C) **Vertical slices through *Thy1-YFP-NL2 *retinas at various ages, showing punctate staining in the IPL. Faint expression in horizontal cells (HCs) was observed transiently during development. INL, inner nuclear layer. Maximal intensity projections of optical planes encompassing 5 to 8 μm thickness.

In order to determine the distribution of inhibitory postsynaptic sites on individual RGC dendritic arbors, we performed our study on a transgenic line in which the *Thy1 *promoter drives expression of NL2 fused to YFP. Retinas from *Tg(Thy1-YFP-NL2) *mice showed punctate expression of YFP-NL2 in a large population of cells in the ganglion cell layer. Bright YFP puncta were present along dendrites of the RGCs, as well as on their somata (Figure 2B). Although many cells in the ganglion cell layer brightly expressed YFP-tagged NL2 in our transgenic line, expression in neurons whose somata located in the inner nuclear layer, including amacrine and bipolar cells, was scarce and very dim (Figure 2C). Thus, not all inner retinal cells containing native NL2 show transgenic expression of YFP-NLG2 in this transgenic line. YFP-NL2 puncta were apparent in the IPL at P8 and localized to this synaptic layer throughout neonatal development and at maturity (Figure 2C). Diffuse intracellular staining was observed in RGCs during early neonatal development but this expression became less apparent by P21. Faint expression was also observed in horizontal cells in the outer retina at P8 but this expression disappeared by P21.

**Figure 3:**
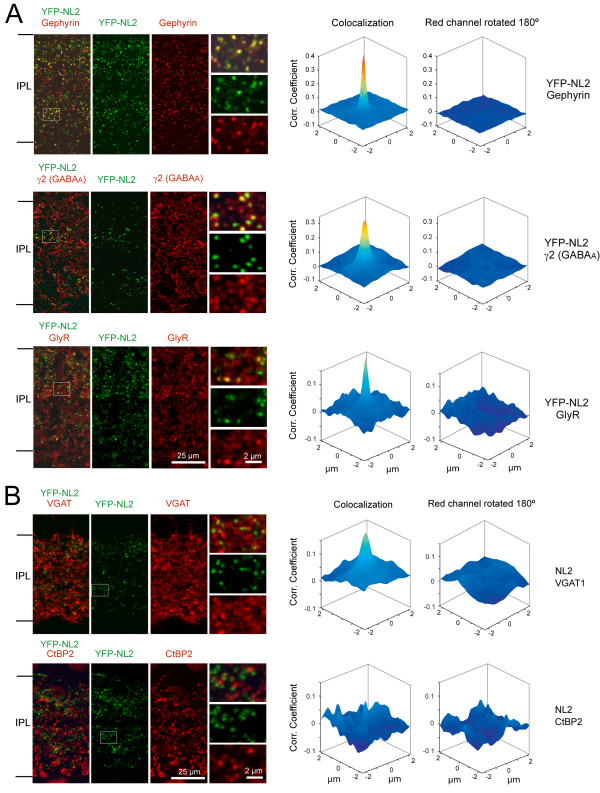
**YFP-NL2 colocalizes with postsynaptic and presynaptic markers of inhibitory synapses in P21 to 24 retinas**. **(A) **Single plane confocal images of vertical slices from *Thy1-YFP-NL2 *retinas labeled with antibodies against the postsynaptic markers gephyrin, the γ2 subunit of GABA_A _receptors and glycine receptors (GlyR). **(B) **YFP-NL2 colocalizes with presynaptic markers of inhibitory but not excitatory synapses. Vesicular GABA and glycine transporter (VGAT) was used as a marker of inhibitory presynaptic sites, and CtBP2 as a marker of excitatory presynaptic sites. Higher magnification views (smaller panels on right) are shown for the boxed regions. For (A, B), two-dimensional cross-correlation coefficient plots of the pixel intensities in the green (YFP-NL2) and red (immunolabeling) channels, and between pixels in the green and rotated red channels, are shown in the right columns.

**Figure 4:**
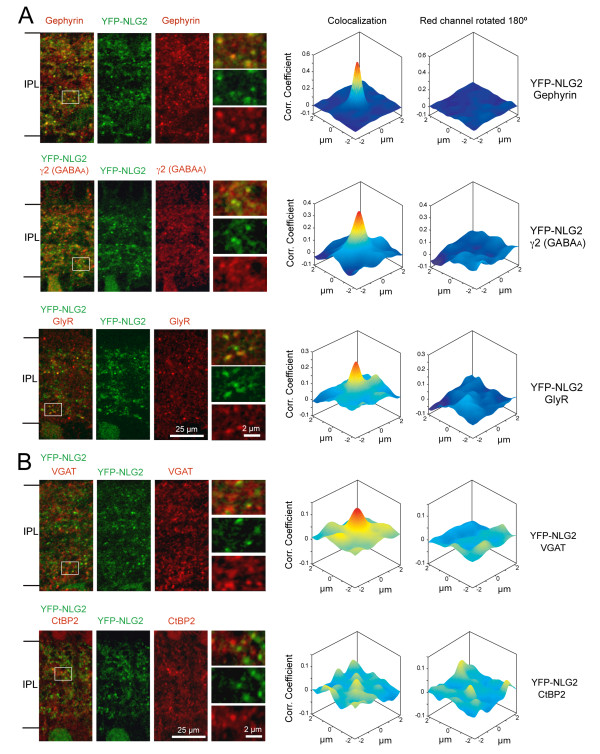
**YFP-NL2 colocalizes with postsynaptic and presynaptic markers of inhibitory synapses at an early developmental age (P10)**. **(A) **Postsynaptic markers. **(B) **Presynaptic marlers. Antibody labeling and cross-correlation analysis were carried out as described for experiments at P21 to P24 (Figure 3). Shown here are single confocal planes of the immunolabeled vibratome sections. Higher magnification views are shown for the boxed regions.

To determine whether the subcellular localization of YFP-NL2 matched the distribution of endogenous NL2 in the mature retina, we first performed immunolabeling for pre- and postsynaptic markers of inhibitory synapses on vertical sections of the transgenic retina at P21 to P24. As observed for endogenous NL2, YFP-NL2 was found to colocalize with gephyrin, the γ2 subunit of GABA_A _receptors and glycine receptors (Figure 3A). In addition, YFP-NL2 clusters were apposed to the inhibitory presynaptic marker, vesicular GABA and glycine transporter (VGAT), but not the presynaptic marker of glutamatergic synapses CtBP2 (Figure 3B). Similarly, immunolabeling for the same set of markers at P10 demonstrates that YFP-NL2 is already appropriately localized to inhibitory synapses at the early stages of bipolar cell synaptogenesis (Figure 4).

### Expression of YFP-NL2 in *Thy1-YFP-NL2 *retina does not alter spontaneous inhibitory postsynaptic currents in RGCs

**Figure 5:**
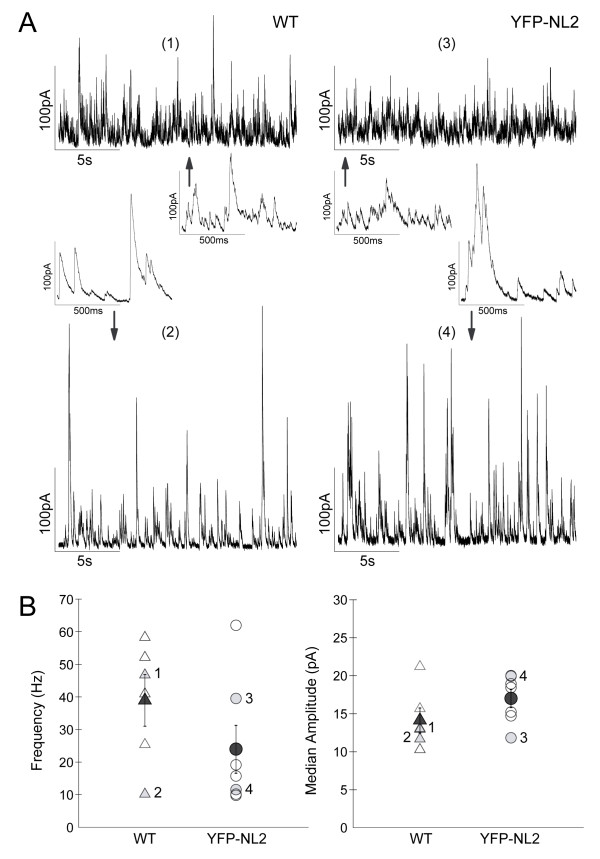
**Expression of YFP-NLG2 does not significantly alter the spontaneous inhibitory drive onto retinal ganglion cells**. **(A) **Example traces showing sIPSCs from whole-cell recordings of wild-type retinas (WT; cells 1 and 2) and YFP-NLG2-expressing retinas (cells 3 and 4) at P21 (inset magnifications showing individual events). **(B) **Mean frequencies and median amplitudes of sIPSCS from WT RGCs (open-triangles, n = 6 cells), and YFP-NLG2-expressing RGCs (open-circles, n = 7 cells). The differences in means between each group (dark grey symols) were not statistically different (frequency *P *= 0.23, amplitude *P *= 0.23, Wilcoxon Rank-sum test). Values for the example traces shown in (A) are indicated by the numbers 1 to 4 (light grey symbols). Error bars are standard error of the means.

Over-expression of NL2 can cause increased localization of NL2 to glutamatergic postsynaptic sites [41]. But, we did not find colocalization of CtBP2 with YFP-NL2 in our transgenic line. Over-expression of NL2 also increases the number of inhibitory synapses in cultured hippocampal neurons [42]. Thus, we performed whole cell patch-clamp experiments and compared the frequency and amplitude of spontaneous inhibitory postsynaptic currents (sIPSCs) of P21 RGCs in YFP-NL2 transgenic retinas (n = 3 retinas) and compared the findings with recordings from wild-type retinas (n = 4 retinas). Our results indicate that there is no significant difference in either the median amplitude or frequency of sIPSCs of YFP-NL2-expressing RGCs when compared to RGCs in wild-type littermates (Figure 5).

### Developmental increases in YFP-NL2 and PSD95-YFP puncta densities on RGC dendrites occur in parallel

**Figure 6:**
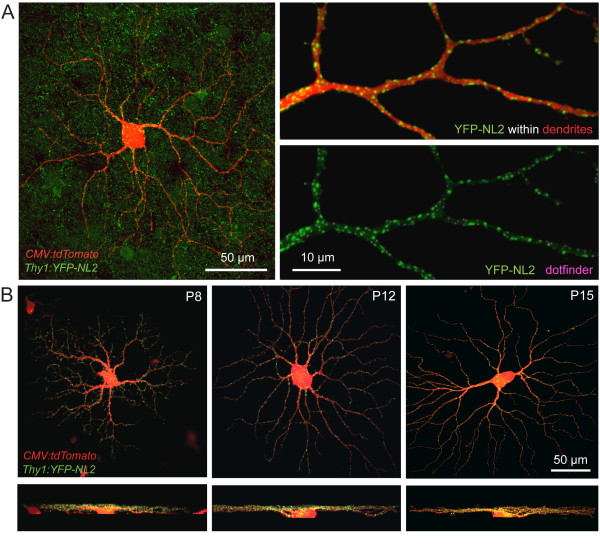
**RGC dendrites and putative inhibitory postsynaptic sites visualized upon expression of tdTomato in RGCs of *Thy1-YFP-NL2 *retina**. **(A) **Whole-mount or *en face *view of a P21 YFP-NL2-expressing (green) ON RGC, co-expressing tdTomato (red). The dendritic labeling was used to perform a 'masking' function that digitally removes YFP-NL2 outside the labeled cell, as shown at high magnification in the right panels. The YFP-NL2 puncta identified using a semi-automated dotfinder program (magenta dots) are superimposed onto the image of the YFP fluorescence. **(B) **Whole-mount view (top) and orthogonal (bottom) projections of large-field ON RGCs at several postnatal ages.

To simultaneously visualize RGC dendrites and inhibitory postsynaptic sites, we biolistically transfected RGCs in YFP-NL2-expressing retinas with a plasmid encoding the red fluorescent protein tdTomato (Figure 6). We restricted our analysis to large-field RGCs whose dendrites stratify in sublamina 'b' of the IPL [43] because these cells were frequently labeled both by the ballistic method and in the transgenic line (Figure 6A). We generated three-dimensional binary masks (Amira, Visage Imaging) of RGC dendrites based on their tdTomato signal. These masks were then used to isolate YFP-NL2 puncta belonging to the tdTomato-labeled cell (Figure 6A, right panels; see Materials and methods).

**Figure 7:**
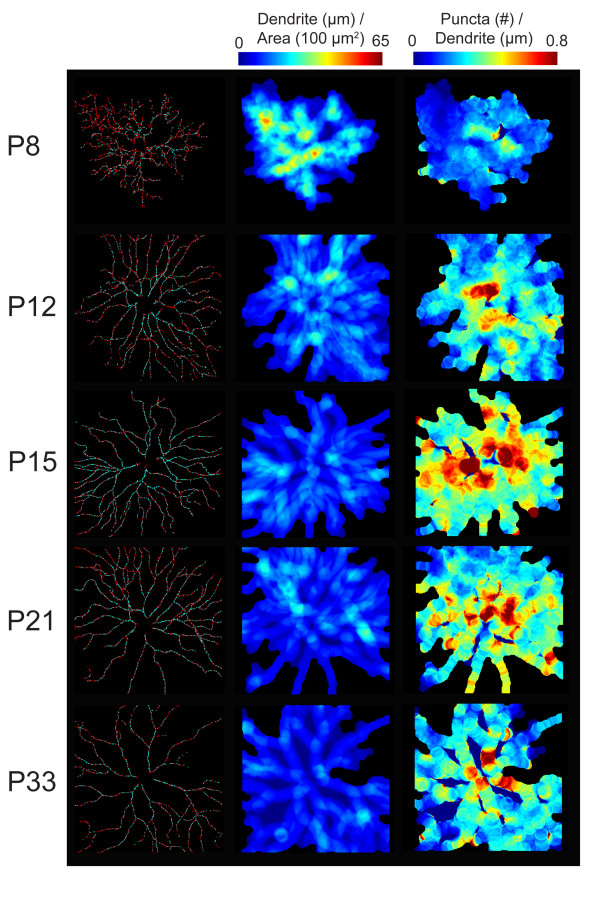
**Spatial maps of dendritic and YFP-NL2 puncta densities of RGCs across development**. Left panels: skeletonization of RGC dendrites (red) and identified YFP-NL2 puncta (blue). Middle panels: dendritic territories determined by convolving a 10 μm diameter disk centered at each pixel with the dendritic skeleton [14]; dendritic density (dendritic length within 100 μm^2 ^area) is mapped across the arbor. Right panels: spatial distributions of YFP-NL2 puncta (linear density; number of puncta per micrometer of dendrite).

Using this approach, we examined the distribution of YFP-NL2 puncta for RGCs at several developmental ages: P7 to P8, during early phase of synapse formation between amacrine cells, bipolar cells and RGCs; P11 to P12, just before eye-opening and when RGCs begin to develop light responses; P15 to P16, around the time of eye-opening; P21, when retinal connections appear established, and P33, when inhibitory and excitatory spontaneous currents attain maturity [44]. Figure 6B shows examples of *en face *and orthogonal views of representative RGCs in wholemount preparations. We then used custom written Matlab routines to obtain the number and distribution of synaptic puncta, as well as total dendritic length and dendritic area [14]. From these parameters, we generated spatial maps of dendritic density (dendritic length divided by dendritic area) and linear density (the number of puncta per unit dendritic length) of YFP-NL2 (Figure 7), similar to what we had charted for PSD95-YFP [14].

**Figure 8:**
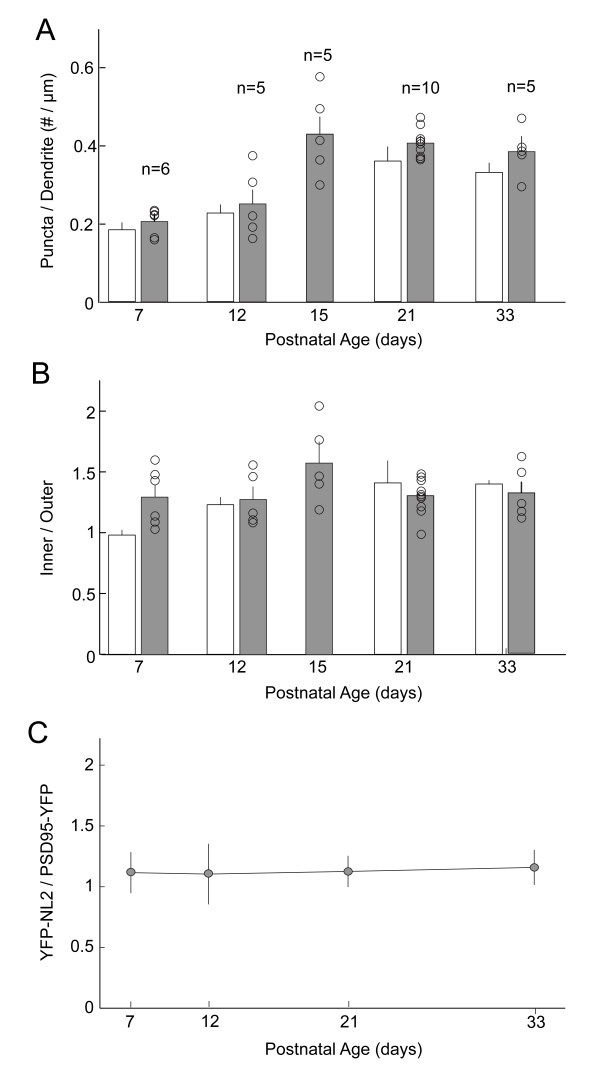
**Inhibitory and excitatory synapse densities on RGC dendrites as a function of age**. **(A) **Linear densities of YFP-NL2 puncta (shaded bars, n = number of cells) compared with the distribution of PSD95 (open bars) during development (data replotted from [14,64]). **(B) **Centro-peripheral gradient of inhibitory and excitatory puncta obtained as described in Morgan *et al*. [14]. The gradients were quantified by determining the ratio of puncta density in the inner and outer halves of a circle encompassing 98% of the dendritic arbor. The analysis excludes a 10-μm region around the cell soma. The inner and outer densities were significantly different at all ages except for P12 (paired *t*-test; *P *< 0.05). **(C) **Ratio of average linear densities of YFP-NL2/PSD95-YFP across ages. Error bars are standard error of the means.

As we showed previously [14], the complexity of RGC arbors, represented by dendritic density, decreased with development. Comparison of the linear density maps across ages suggested that YFP-NL2 puncta density increased with maturation (Figure 7). Quantification across cells confirmed this impression, showing that, on average, YFP-NL2 linear density increased until around P15, whereupon it remained relatively unchanged (Figure 8A). When we compared YFP-NL2 linear density within the inner and outer halves of the dendritic arbor as a function of age, we found that YFP-NL2 densities were higher in central compared to the peripheral parts of the arbor, and that this gradient was already apparent at the earliest ages studied, P7 to P8 (Figure 8B).

Finally, we compared the developmental patterning of YFP-NL2 with the distribution of excitatory postsynaptic sites, marked by PSD95-YFP expression, on large-field ON-type RGCs that stratify in the inner part of the IPL. It is evident in Figure 8A, B that both PSD95-YFP and YFP-NL2 puncta densities increased in parallel with age. The average ratio of YFP-NL2/PSD95-YFP puncta densities per RGC was relatively unchanged from P7 until maturity (Figure 8C). Thus, the mature ratio (1.16 ± 0.14; P33) of inhibitory to excitatory synapse number onto large-field ON RGCs is attained shortly after of the onset of glutamatergic synaptogenesis, and several weeks before inner retinal circuits are mature.

## Discussion

### YFP-tagged NL2 expression resembles endogenous NL2 expression

NL2 is one of the earliest components of the postsynaptic specialization of inhibitory synapses, and helps recruit gephyrin [34,45] and GABA_A _receptors to these sites [36,40]. In the retina of *Thy1-YFP-NL2 *transgenic mice, punctate distribution of YFP-NL2 in the IPL resembles clustering of endogenous NL2 [40,46]. Like endogenous NL2 [40], YFP-NL2 colocalized with the γ2 subunit of the GABA_A _receptor, a key component of synaptic GABA_A _receptors [47]. In the retinal IPL, gephyrin is mainly associated with a subset of GABA_A _(α2-containing) receptors [48] and is present in all glycinergic synapses, with the exception of connections onto rod bipolar cell terminals [37]. We observed co-localization of NL2 with gephyrin that corroborates previous reports of a direct interaction between these two proteins [45]. This, together with the fact that we also found co-localization of NL2 puncta with glycine receptor subunits, indicates that YFP-NL2 is also present at glycinergic postsynaptic specializations. In addition, we observed appositions with a presynaptic marker of inhibitory (VGAT) but not excitatory (CtBP2) synapses. YFP-NL2 therefore appears to be a suitable marker of GABAergic and glycinergic inhibitory postsynapses in the mouse retinal IPL.

One concern that we had was that in our transgenic line, expression of YFP-NL2 may alter synapse number because NL2 over-expression increases the number of both inhibitory and excitatory synaptic connections [41]. However, we found that RGCs in *Thy1-YFP-NL2 *mice showed no significant change in the median amplitude and frequency of spontaneous inhibitory synaptic currents. Moreover, transgenic YFP-NL2 animals showed grossly normal behavioral traits, life spans and offspring production. This is in contrast to what was described for transgenic lines with high levels of NL2 over-expression (50% or more above endogenous levels), which displayed a variety of phenotypes, including altered synaptic function, early postnatal death, and behavioral changes such as limb clasping, repetitive behavior and anxiety [49]. Thus, we believe that the distribution of NL2 is not grossly over-expressed in our transgenic line, and YFP-NL2 puncta across the dendritic arbors of the RGCs likely reflect endogenous distributions.

### Spatial maps of YFP-NL2 puncta for mature large-field ON RGCs

To date, the densities and distribution patterns of amacrine synapses onto the dendrites of RGCs in the mature retina have been obtained largely by serial EM. As such, only a handful of cells can be fully reconstructed. More recently, combining immunostaining for transmitter receptors with single cell labeling has enabled comparisons of the distributions of excitatory and inhibitory synapses on adult primate RGCs [27,28]. Here, we used the expression of YFP-NL2 to mark the locations of inhibitory postsynaptic sites and revealed the spatial distributions of amacrine synapses on the dendritic arbors of large field ON RGCs. One month postnatally, we found that, on average, the linear density of YFP-NL2 is 0.38 ± 0.03 puncta/μm. This density is similar to the density of amacrine synapses on the dendrites of a large-field ON alpha-RGC in the adult cat retina (0.36 synapse/μm), previously reconstructed by EM [22].

Serial EM and partial three-dimensional reconstructions of RGC dendritic trees in the cat, marmoset and rabbit retina have also revealed a considerable variation in the ratio of inhibitory and excitatory synapses onto individual RGCs [20-24,50]. Amacrine synapses comprise 50 to 80% of the total number of synapses on a RGC, depending on the species and the type of RGC analyzed. Confocal reconstructions of rabbit direction-selective RGCs in which presynaptic ribbon-associated terminals were labeled and inhibitory postsynaptic sites revealed by immunostaining for the α1 subunit of the GABA_A _receptor suggest that inhibitory synapses constitute at least one-third of all synapses onto these RGCs [21,50]. In our study, comparison between NL2 and PSD95 puncta densities suggests that amacrine and bipolar cells form almost equal numbers of synapses on mature large-field mouse ON RGCs. Additionally, we found that there is a shallow gradient (about 1.5) of NL2 puncta density from the center to peripheral parts of the dendritic arbor of the large-field RGCs, similar to the gradient of glutamatergic postsynaptic sites on these RGCs [14]. Thus, the densities of inhibitory and excitatory postsynaptic sites appear matched across the dendritic arbor of these mouse RGCs.

### Coordinated development of inhibitory and excitatory postsynaptic sites onto RGCs

It is evident from morphological and physiological studies that, *in vivo*, GABAergic and glycinergic synaptic connections are formed before glutamatergic connections. For example, in the hippocampus, the apical dendrite of CA1 pyramidal cells is first contacted by GABAergic inteneurons prior to extension of these dendrites into the stratum lacunosum-moleculare, where they later receive input from glutamatergic afferents of the perforant pathway [4,5]. Likewise, RGCs also obtain GABAergic and glycinergic drive from amacrine cells prior to the formation of glutamatergic synapses from bipolar cells [51] (but see [52]). Unlike hippocampal pyramidal cells where dendrites need to reach available glutamatergic afferents, the delay in glutamatergic synaptogenesis onto RGCs is because bipolar cells are generated much later than amacrine cells. As apparent in other model systems, GABAergic and glycinergic transmission onto RGCs is also initially depolarizing, but switches to hyperpolarizing when glutamatergic synapses are established [53-55]. Thus, circuitry of the inner retina of vertebrates is a good representative model for comparing the development of inhibitory versus excitatory synapses onto the same postsynaptic cell type. By mapping the distribution and density of YFP-NL2 on the dendrites of individually labeled large-field RGCs, we found that inhibitory synaptogenesis onto these cells peaks at the end of the second postnatal week, and stays relatively constant thereafter. This temporal profile in the generation of amacrine synapses matches that previously suggested by following changes in total inhibitory synapse density across the mouse IPL [29]. Rapid amacrine synaptogenesis during the first and second postnatal weeks also appears to occur in other species, as revealed by immunostaining for GABA_A _receptors, glycine receptor subunits or gephyrin on RGCs, for example in rat and rabbit retina [37,56].

Our observations here show that the time course in the increase in YFP-NL2 puncta density with age parallels that of PSD95-YFP on the dendrites of large-field RGCs [14], resulting in a surprisingly constant inhibitory/excitatory synapse ratio across the RGC arbor shortly after bipolar cell synaptogenesis commences. This inhibitory to excitatory synapse ratio appears maintained throughout neonatal development despite significant dendritic remodeling [14] and large-scale synaptogenesis during this period [29]. The increase in YFP-NL2 and PSD95-YFP puncta densities may also be coordinated locally on the dendritic arbor as well as across ages because the centro-peripheral gradients of both YFP-NL2 and PSD95-YFP appeared by P12 [14] and persist into adulthood. Indeed, in hippocampal neurons in culture, the ratio of excitatory and inhibitory synapses is matched at the level of individual dendritic branches, producing a local balance of excitation and inhibition [57]. However, unlike our current findings for RGCs, this ratio for hippocampal neurons increases between the second and third week *in vitro *[57,58]. It should be noted, however, that although the ratio of inhibitory to excitatory synapses appears constant across ages for the large-field ON RGCs, functional drive from bipolar cells and amacrine cells onto RGCs increases with maturation. Whole-cell recordings from neonatal mouse retina show that both spontaneous inhibitory and excitatory postsynaptic current frequencies increase concurrently over the first postnatal month [59].

What mechanisms regulate the ratio of inhibitory to excitatory synapses on dendrites? It is likely that apart from glutamatergic transmission [60,61], GABAergic transmission itself plays a role in regulating the balance of inhibitory and excitatory inputs in developing CNS circuits [62,63]. Glutamatergic transmission certainly regulates the number of synapses formed between bipolar cells and the large-field RGCs [64], but as yet, we do not know how amacrine synapse numbers or distributions on these RGCs might be affected. Conversely, the influence of GABAergic transmission on amacrine or bipolar cell synaptogenesis on to RGC dendrites has yet to be explored. It is evident, however, that whatever the role neurotransmission plays in setting up amacrine-bipolar cell synaptic ratios, the final synapse numbers from each input type are likely to be shaped by the relative addition and elimination of synapses [64]. Currently, we do not know whether, like bipolar cell contacts, amacrine connections also undergo remodeling during development, although it is possible given that amacrine neurites show structural rearrangements during the period of synaptogenesis [65]. A further consideration is that glycinergic amacrine cells are born later than GABAergic amacrine cells [66,67]. Because NL2 does not necessarily distinguish GABAergic from glycinergic postsynaptic sites in the retinal IPL, future studies specifically marking GABAergic or glycinergic synapses will help distinguish the contributions of these two major amacrine cell populations to the observed increase in conventional synapses during postnatal development. Such knowledge will help provide further insight into how the development of amacrine and bipolar cell connectivity onto RGCs is coordinated.

## Conclusions

Based on YFP-NL2 expression in a *Thy1-YFP-NL2 *transgenic mouse line we generated, we found that the density of inhibitory amacrine synapses on the arbors of large-field ON RGCs increased gradually, until around eye opening (P15). By comparing the spatial densities of YFP-NL2 with PSD95-YFP across the dendritic arbors of these RGCs, we discovered that their adult ratio of inhibitory/excitatory postsynaptic sites was rapidly attained, shortly after bipolar cells form synapses in the IPL (P7). Our observations suggest that bipolar and amacrine cell synaptogenesis onto RGCs are coordinated, and a balanced ratio of excitatory and inhibitory synapse densities is established prior to the onset of visual experience.

## Materials and methods

### Generation of *Thy1-YFP-NL2 *transgenic mice

YFP-NL2 expressed here was modified from Graf *et al*. [34] and consists of the signal sequence of mouse Neuroligin-1, hexahistidine (HHHHHH), Flag (GGDYKDDDDK), and EYFP tags followed by the mature coding sequence of mouse NL2. The *Thy1 *promoter [68] was used to drive expression of YFP-NL2. The transgene was generated by cloning the *Thy1 *promoter fragment into shuttle vector LNL, which contained the needed restriction sites for transgene release. YFP-NL2 was digested with HindIII and Afl II and blunted into XhoI cut and blunted thy1-LNL. The *Thy1-YFP-NL2 *transgene was released from the vector backbone sequence by restriction digestion with AscI and PmeI and injected into B6/CBA F1 hybrid pronuclei to generate founder mice.

### Immunohistochemistry

C57BL/6 (P21) and *Thy1-YFP-NL2 *(P7, P12, P15, P21) mice were deeply anesthetized with 5% isofluorane and decapitated. Eyes were removed and placed in ice cold mouse artificial cerebrospinal fluid (mACSF; 119 mM NaCl, 2.5 mM KCl, 2.5 mM CaCl_2_, 1.3 mM MgCl_2_, 1 mM NaH_2_PO_4_, 11 mM glucose (20), 20 mM HEPES, pH = 7.4). After removing the lens and vitreous, the eye cup was fixed in 4% paraformaldehyde for 15 to 30 minutes. After fixation, the eye cups were rinsed in 0.1 M PBS. The retina was removed from the eye cup, embedded in 4% low-melting point agarose and cut into 60 μm thick sections using a vibratome. Sections were mounted and used for imaging or processed for immunostaining as follows: blocked in 10% NGS in PBS for 1 hour followed by overnight incubation in 5% NGS, 0.5% Triton-X100, with the corresponding primary antibodies. The primary antibodies used were: rabbit anti-NL2 antibody (1:8,000; generous gift of F Varoqueaux and N Brose) [35,40], guinea-pig anti-γ2 antibody (1:1,000; generous gift of JM Fritschy), a monoclonal mouse antibody (mAb4a (P21) against all glycine receptor subunits,1:400; or mAb2b (P10), against the α1 subunit of the glycine receptor, 1:400; Synaptic Systems, Goettingen, Germany), anti-gephyrin mAb7a (1:500; Synaptic Systems), anti-VGAT antibody (1:1,000; Millipore, Temecula, CA, USA), anti-CtBP2 antibody (1:1000; BD Transduction, Franklin Lakes, NJ, USA). Sections were then washed and incubated for 1 hour with the corresponding secondary antibody conjugated to either Alexa-488 or Alexa-568 (1:1,000; Invitrogen, Carlsbad, CA, USA).

### Cell transfection

*Thy1-YFP-NL2 *mice were deeply anesthetized with 5% isofluorane and decapitated. Eyes were removed and placed in ice cold mouse mACSF. Retinas were removed from the eye cup and mounted RGC side up on black nitrocellulose filter paper (HABP013, Millipore, Bedford, MA, USA). Gold particles were coated with 20 μg *CMV:tdtomato *DNA (gift of R Tsien) and delivered using a Bio-Rad Helios gene-gun as previously described [14]. Retinas were incubated for 18 to 24 hours at 33°C, fixed for 30 minutes in 4% parafolmaldehyde in mACSF, washed in PBS and mounted in Vectashield (Vector Labs, Burlingame, CA, USA). The data presented in this study were obtained from six P8, five P12, five P15, ten P21 and five P30 RGCs.

### Imaging and image analysis

Images were obtained using a 1.35 NA 60× oil objective (Olympus). Images were acquired at 0.069 × 0.069 × 0.3 μm for double labeling immunohistochemistry (Figures 1, 3 and 4), and 0.103 × 0.103 × 0.3 μm voxel sizes for vertical slices of YFP-NL2 retinas (Figure 2) and retinal whole-mounts (Figures 2 and 6). Images were processed using Metamorph (Molecular Devices, Sunnyvale, CA, USA), Image J (NCBI), Amira (Mercury Computer Systems Inc., Chelmsford, MA, USA) and Matlab (Math Works, Natick, MA, USA). Images were median-filtered to reduce noise. The contrast and gamma of the images were adjusted to increase visualization of dim objects. Using the 'label-field' function of the AMIRA software program, a threshold was applied, plane by plane, to capture pixels representing the dendrites of the RGCs [14]. This procedure generated a binary mask of the dendrites that was then used to isolate puncta from the YFP-NL2 channel that resided within the mask (YFP-NL2-labeled voxels outside the mask were then discarded). In the same process, cell somas were removed before further analysis. Custom Matlab programs were used to generate dendritic skeletons and to identify puncta, dendritic lengths and dendritic areas as previously described [14]. Dendritic density represents the total dendritic length divided by the total area of the dendritic territory.

In order to assess whether NL2 signal significantly overlaps with other synapse markers, a custom Matlab program was used to calculate the two-dimensional cross-correlation coefficients of the signals from both channels (Josh Morgan and Daniel Kerschensteiner). This approach does not require identification of puncta, yet allows us to determine whether the fluorescent signals in the two channels are spatially correlated, or are randomly associated (random association determined by rotating one image 180° relative to the other image).

Within an optical plane, the signal intensity of a pixel in the green channel was compared with the signal intensity of the corresponding pixel in the red channel (0,0 location). To obtain the two-dimensional correlation plot, the intensity of a green pixel was compared with the intensity of red pixels (or vice versa) to the right, left, top and bottom, displaced from the reference pixel up to 2.8 μm. A pixel intensity threshold of 20 to 35, defining the background, was used while analyzing images obtained from wild-type mouse retina. Thresholding was not used when analyzing images obtained from the YFP-NL2 transgenic mouse line due to the lower immunohistochemistry background level in the transgenic retina.

Representative examples shown in the figures are cross-correlation coefficients calculated based on 20 to 45 image planes (z-step size = 0.3 μm) of an imaged field of view. The equation used to calculate the correlation coefficient is:

$$R\left(r,g\right)=\frac{C\left(r,g\right)}{\sqrt{C\left(r,r\right),C\left(g,g\right)}}$$

where R(r, g) is the correlation coefficient of the red and green channel and C is the covariance of the corresponding channels.

For two identical images, the correlogram peak is 1. The value of the positive peak for two identical images is only weakly influenced by the absolute intensities of the pixels within an image but strongly corresponds to how spatially 'similar' the two images are. For overlapping green and red puncta, the peak of the two-dimensional correlation plot falls off symmetrically on all sides. The correlograms with positive peaks have a full-width at half maximum of less than 1 μm, suggesting that there exist structures in the two images within less than 1 μm overlap in space.

### Electrophysiology

Retinal flat mounts were prepared as described above for cell transfection, but mounted on white filter paper (Anodisc 13, Whatman Inc., Piscataway, NJ, USA) for better visualization. Recordings were performed at room temperature and retinas were maintained in bicarbonate-buffered mACSF containing 125 mM NaCl, 2.5 mM KCl, 2 mM CaCl_2_, 1 mM MgCl_2_, 1.25 mM NaH_2_PO_4_, 11 mM glucose and 26 mM NaHCO_3 _(equilibrated with 95% O_2 _and 5% CO_2_). Whole-cell recordings were performed with electrodes (4 to 8 MΩ) filled with 120 mM Cs-gluconate, 1 mM CaCl_2_, 1 mM MgCl_2_, 10 mM Na-HEPES, 11 mM EGTA, and 10 mM TEA-Cl (pH 7.2 adjusted with CsOH). For some experiments 2 mM QX314 was also included in the patch pipette. A liquid junction potential of 15 mV was corrected before the cell was attached, and series resistance was not compensated. Data were acquired using an Axopatch 200 B amplifier (Molecular Devices), low-pass filtered at 2 kHz and digitized at 5 kHz. sIPSCs were recorded at -0 mV, the reversal potential of cation currents in our recording conditions. Area and amplitude thresholds (Mini Analysis, Synaptosoft, Decatur, GA, USA) were optimized to detect > 90% of the events identified by eye for the entirety of recordings analyzed. For overlapping events, the baseline for amplitude measurement of each event was estimated from exponential decay extrapolation of the previous event.

## Abbreviations

CNS: central nervous system; CtBP2: carboxy-terminal binding protein 2; EM: electron microscopy; GABA: γ-aminobutyric acid; IPL: inner plexiform layer; mACSF: mouse artificial cerebrospinal fluid; NL2: Neuroligin 2; P: postnatal day; PBS: phosphate-buffered saline; RGC: retinal ganglion cell; sIPSC: spontaneous inhibitory postsynaptic current; VGAT: vesicular inhibitory transmitter transporter; YFP: yellow fluorescent protein.

## Competing interests

The authors declare that they have no competing interests.

## Authors' contributions

FS and AB carried out the experiments. FS, AB and DK performed the analysis. RL, YK, and AMC generated the transgenic mice. FS and ROLW designed and coordinated the work. All authors read and approved the final manuscript.

## References

[B1] Salinas E Sejnowski TJ Impact of correlated synaptic input on output firing rate and variability in simple neuronal models J Neurosci 2000 20 6193 6209 10934269 10.1523/JNEUROSCI.20-16-06193.2000 PMC6772574

[B2] Turrigiano GG The self-tuning neuron: synaptic scaling of excitatory synapses Cell 2008 135 422 435 10.1016/j.cell.2008.10.008 18984155 PMC2834419

[B3] Feldman DE Synaptic mechanisms for plasticity in neocortex Annu Rev Neurosci 2009 32 33 55 10.1146/annurev.neuro.051508.135516 19400721 PMC3071739

[B4] Hennou S Khalilov I Diabira D Ben-Ari Y Gozlan H Early sequential formation of functional GABA(A) and glutamatergic synapses on CA1 interneurons of the rat foetal hippocampus Eur J Neurosci 2002 16 197 208 10.1046/j.1460-9568.2002.02073.x 12169102

[B5] Tyzio R Represa A Jorquera I Ben-Ari Y Gozlan H Aniksztejn L The establishment of GABAergic and glutamatergic synapses on CA1 pyramidal neurons is sequential and correlates with the development of the apical dendrite J Neurosci 1999 19 10372 10382 10575034 10.1523/JNEUROSCI.19-23-10372.1999 PMC6782402

[B6] Alvarez VA Sabatini BL Anatomical and physiological plasticity of dendritic spines Annu Rev Neurosci 2007 30 79 97 10.1146/annurev.neuro.30.051606.094222 17280523

[B7] Bourne JN Harris KM Balancing structure and function at hippocampal dendritic spines Annu Rev Neurosci 2008 31 47 67 10.1146/annurev.neuro.31.060407.125646 18284372 PMC2561948

[B8] Holtmaat A Svoboda K Experience-dependent structural synaptic plasticity in the mammalian brain Nat Rev Neurosci 2009 10 647 658 10.1038/nrn2699 19693029

[B9] Pan F Gan WB Two-photon imaging of dendritic spine development in the mouse cortex Dev Neurobiol 2008 68 771 778 10.1002/dneu.20630 18383548

[B10] Erikoz B Jusuf PR Percival KA Grunert U Distribution of bipolar input to midget and parasol ganglion cells in marmoset retina Vis Neurosci 2008 25 67 76 18282311 10.1017/S0952523808080073

[B11] Jakobs TC Koizumi A Masland RH The spatial distribution of glutamatergic inputs to dendrites of retinal ganglion cells J Comp Neurol 2008 510 221 236 10.1002/cne.21795 18623177 PMC2566960

[B12] Koizumi A Jakobs TC Masland RH Regular mosaic of synaptic contacts among three retinal neurons J Comp Neurol 2011 519 341 357 10.1002/cne.22522 21165978 PMC3140001

[B13] Lin B Martin PR Grunert U Expression and distribution of ionotropic glutamate receptor subunits on parasol ganglion cells in the primate retina Vis Neurosci 2002 19 453 465 12511078 10.1017/s0952523802194077

[B14] Morgan JL Schubert T Wong RO Developmental patterning of glutamatergic synapses onto retinal ganglion cells Neural Dev 2008 3 8 10.1186/1749-8104-3-8 18366789 PMC2311295

[B15] Niell CM Meyer MP Smith SJ *In vivo *imaging of synapse formation on a growing dendritic arbor Nat Neurosci 2004 7 254 260 10.1038/nn1191 14758365

[B16] Huang ZJ Activity-dependent development of inhibitory synapses and innervation pattern: role of GABA signalling and beyond J Physiol 2009 587 1881 1888 10.1113/jphysiol.2008.168211 19188247 PMC2689329

[B17] Megias M Emri Z Freund TF Gulyas AI Total number and distribution of inhibitory and excitatory synapses on hippocampal CA1 pyramidal cells Neuroscience 2001 102 527 540 10.1016/S0306-4522(00)00496-6 11226691

[B18] MacNeil MA Masland RH Extreme diversity among amacrine cells: implications for function Neuron 1998 20 971 982 10.1016/S0896-6273(00)80478-X 9620701

[B19] Wassle H Koulen P Brandstatter JH Fletcher EL Becker CM Glycine and GABA receptors in the mammalian retina Vision Res 1998 38 1411 1430 10.1016/S0042-6989(97)00300-3 9667008

[B20] Dacheux RF Chimento MF Amthor FR Synaptic input to the on-off directionally selective ganglion cell in the rabbit retina J Comp Neurol 2003 456 267 278 10.1002/cne.10521 12528191

[B21] Famiglietti EV Synaptic organization of complex ganglion cells in rabbit retina: type and arrangement of inputs to directionally selective and local-edge-detector cells J Comp Neurol 2005 484 357 391 10.1002/cne.20433 15770656

[B22] Freed MA Sterling P The ON-alpha ganglion cell of the cat retina and its presynaptic cell types J Neurosci 1988 8 2303 2320 3249227 10.1523/JNEUROSCI.08-07-02303.1988 PMC6569538

[B23] Ghosh KK Grunert U Synaptic input to small bistratified (blue-ON) ganglion cells in the retina of a new world monkey, the marmoset *Callithrix jacchus* J Comp Neurol 1999 413 417 428 10.1002/(SICI)1096-9861(19991025)413:3<417::AID-CNE5>3.0.CO;2-H 10502249

[B24] Haverkamp S Eldred WD Ottersen OP Pow D Ammermuller J Synaptic inputs to identified color-coded amacrine and ganglion cells in the turtle retina J Comp Neurol 1997 389 235 248 10.1002/(SICI)1096-9861(19971215)389:2<235::AID-CNE4>3.0.CO;2-2 9416919

[B25] Cohen E Sterling P Parallel circuits from cones to the on-beta ganglion cell Eur J Neurosci 1992 4 506 520 10.1111/j.1460-9568.1992.tb00901.x 12106337

[B26] Kolb H Nelson R OFF-alpha and OFF-beta ganglion cells in cat retina: II. Neural circuitry as revealed by electron microscopy of HRP stains J Comp Neurol 1993 329 85 110 10.1002/cne.903290107 8454727

[B27] Percival KA Jusuf PR Martin PR Grunert U Synaptic inputs onto small bistratified (blue-ON/yellow-OFF) ganglion cells in marmoset retina J Comp Neurol 2009 517 655 669 10.1002/cne.22183 19830807

[B28] Percival KA Martin PR Grunert U Synaptic inputs to two types of koniocellular pathway ganglion cells in marmoset retina J Comp Neurol 2011 519 2135 2153 10.1002/cne.22586 21452222

[B29] Fisher LJ Easter SS Jr Retinal synaptic arrays: continuing development in the adult goldfish J Comp Neurol 1979 185 373 379 10.1002/cne.901850210 429621

[B30] Schubert T Kerschensteiner D Eggers ED Misgeld T Kerschensteiner M Lichtman JW Lukasiewicz PD Wong RO Development of presynaptic inhibition onto retinal bipolar cell axon terminals is subclass-specific J Neurophysiol 2008 100 304 316 10.1152/jn.90202.2008 18436633 PMC2493474

[B31] Nishimura Y Rakic P Development of the rhesus monkey retina: II. A three-dimensional analysis of the sequences of synaptic combinations in the inner plexiform layer J Comp Neurol 1987 262 290 313 10.1002/cne.902620209 3624556

[B32] Crooks J Okada M Hendrickson AE Quantitative analysis of synaptogenesis in the inner plexiform layer of macaque monkey fovea J Comp Neurol 1995 360 349 362 10.1002/cne.903600211 8522652

[B33] Varoqueaux F Aramuni G Rawson RL Mohrmann R Missler M Gottmann K Zhang W Sudhof TC Brose N Neuroligins determine synapse maturation and function Neuron 2006 51 741 754 10.1016/j.neuron.2006.09.003 16982420

[B34] Graf ER Zhang X Jin SX Linhoff MW Craig AM Neurexins induce differentiation of GABA and glutamate postsynaptic specializations via neuroligins Cell 2004 119 1013 1026 10.1016/j.cell.2004.11.035 15620359 PMC2826211

[B35] Varoqueaux F Jamain S Brose N Neuroligin 2 is exclusively localized to inhibitory synapses Eur J Cell Biol 2004 83 449 456 10.1078/0171-9335-00410 15540461

[B36] Patrizi A Scelfo B Viltono L Briatore F Fukaya M Watanabe M Strata P Varoqueaux F Brose N Fritschy JM Sassoe-Pognetto M Synapse formation and clustering of neuroligin-2 in the absence of GABAA receptors Proc Natl Acad Sci USA 2008 105 13151 13156 10.1073/pnas.0802390105 18723687 PMC2529038

[B37] Sassoe-Pognetto M Wassle H Synaptogenesis in the rat retina: subcellular localization of glycine receptors, GABA(A) receptors, and the anchoring protein gephyrin J Comp Neurol 1997 381 158 174 10.1002/(SICI)1096-9861(19970505)381:2<158::AID-CNE4>3.0.CO;2-2 9130666

[B38] Fritschy JM Mohler H GABAA-receptor heterogeneity in the adult rat brain: differential regional and cellular distribution of seven major subunits J Comp Neurol 1995 359 154 194 10.1002/cne.903590111 8557845

[B39] Bechade C Colin I Kirsch J Betz H Triller A Expression of glycine receptor alpha subunits and gephyrin in cultured spinal neurons Eur J Neurosci 1996 8 429 435 10.1111/j.1460-9568.1996.tb01226.x 8714713

[B40] Hoon M Bauer G Fritschy JM Moser T Falkenburger BH Varoqueaux F Neuroligin 2 controls the maturation of GABAergic synapses and information processing in the retina J Neurosci 2009 29 8039 8050 10.1523/JNEUROSCI.0534-09.2009 19553444 PMC6666037

[B41] Levinson JN El-Husseini A Building excitatory and inhibitory synapses: balancing neuroligin partnerships Neuron 2005 48 171 174 10.1016/j.neuron.2005.09.017 16242398

[B42] Chubykin AA Atasoy D Etherton MR Brose N Kavalali ET Gibson JR Sudhof TC Activity-dependent validation of excitatory versus inhibitory synapses by neuroligin-1 versus neuroligin-2 Neuron 2007 54 919 931 10.1016/j.neuron.2007.05.029 17582332 PMC3738748

[B43] Volgyi B Chheda S Bloomfield SA Tracer coupling patterns of the ganglion cell subtypes in the mouse retina J Comp Neurol 2009 512 664 687 10.1002/cne.21912 19051243 PMC3373319

[B44] Johnson J Tian N Caywood MS Reimer RJ Edwards RH Copenhagen DR Vesicular neurotransmitter transporter expression in developing postnatal rodent retina: GABA and glycine precede glutamate J Neurosci 2003 23 518 529 12533612 10.1523/JNEUROSCI.23-02-00518.2003 PMC6741860

[B45] Poulopoulos A Aramuni G Meyer G Soykan T Hoon M Papadopoulos T Zhang M Paarmann I Fuchs C Harvey K Jedlicka P Schwarzacher SW Betz H Harvey RJ Brose N Zhang W Varoqueaux F Neuroligin 2 drives postsynaptic assembly at perisomatic inhibitory synapses through gephyrin and collybistin Neuron 2009 63 628 642 10.1016/j.neuron.2009.08.023 19755106

[B46] Koulen P Sassoe-Pognetto M Grunert U Wassle H Selective clustering of GABA(A) and glycine receptors in the mammalian retina J Neurosci 1996 16 2127 2140 8604056 10.1523/JNEUROSCI.16-06-02127.1996 PMC6578501

[B47] Essrich C Lorez M Benson JA Fritschy JM Luscher B Postsynaptic clustering of major GABAA receptor subtypes requires the gamma 2 subunit and gephyrin Nat Neurosci 1998 1 563 571 10.1038/2798 10196563

[B48] Sassoe-Pognetto M Kirsch J Grunert U Greferath U Fritschy JM Mohler H Betz H Wassle H Colocalization of gephyrin and GABAA-receptor subunits in the rat retina J Comp Neurol 1995 357 1 14 10.1002/cne.903570102 7673460

[B49] Hines RM Wu L Hines DJ Steenland H Mansour S Dahlhaus R Singaraja RR Cao X Sammler E Hormuzdi SG Zhuo M El-Husseini A Synaptic imbalance, stereotypies, and impaired social interactions in mice with altered neuroligin 2 expression J Neurosci 2008 28 6055 6067 10.1523/JNEUROSCI.0032-08.2008 18550748 PMC6670530

[B50] Jeon CJ Kong JH Strettoi E Rockhill R Stasheff SF Masland RH Pattern of synaptic excitation and inhibition upon direction-selective retinal ganglion cells J Comp Neurol 2002 449 195 205 10.1002/cne.10288 12115689

[B51] Unsoeld T Stradomska AM Wang R Rathjen FG Juttner R Early maturation of GABAergic synapses in mouse retinal ganglion cells Int J Dev Neurosci 2008 26 233 238 10.1016/j.ijdevneu.2007.12.001 18207351

[B52] Rorig B Grantyn R Rat retinal ganglion cells express Ca(2+)-permeable non-NMDA glutamate receptors during the period of histogenetic cell death Neurosci Lett 1993 153 32 36 10.1016/0304-3940(93)90070-2 8510821

[B53] Leitch E Coaker J Young C Mehta V Sernagor E GABA type-A activity controls its own developmental polarity switch in the maturing retina J Neurosci 2005 25 4801 4805 10.1523/JNEUROSCI.0172-05.2005 15888655 PMC6724776

[B54] Vu TQ Payne JA Copenhagen DR Localization and developmental expression patterns of the neuronal K-Cl cotransporter (KCC2) in the rat retina J Neurosci 2000 20 1414 1423 10662832 10.1523/JNEUROSCI.20-04-01414.2000 PMC6772353

[B55] Zhang LL Pathak HR Coulter DA Freed MA Vardi N Shift of intracellular chloride concentration in ganglion and amacrine cells of developing mouse retina J Neurophysiol 2006 95 2404 2416 16371454 10.1152/jn.00578.2005

[B56] Koulen P Postnatal development of GABAA receptor beta1, beta2/3, and gamma2 immunoreactivity in the rat retina J Neurosci Res 1999 57 185 194 10.1002/(SICI)1097-4547(19990715)57:2<185::AID-JNR4>3.0.CO;2-T 10398296

[B57] Liu G Local structural balance and functional interaction of excitatory and inhibitory synapses in hippocampal dendrites Nat Neurosci 2004 7 373 379 10.1038/nn1206 15004561

[B58] Benson DL Cohen PA Activity-independent segregation of excitatory and inhibitory synaptic terminals in cultured hippocampal neurons J Neurosci 1996 16 6424 6432 8815921 10.1523/JNEUROSCI.16-20-06424.1996 PMC6578921

[B59] Tian N Copenhagen DR Visual deprivation alters development of synaptic function in inner retina after eye opening Neuron 2001 32 439 449 10.1016/S0896-6273(01)00470-6 11709155

[B60] Colin-Le Brun I Ferrand N Caillard O Tosetti P Ben-Ari Y Gaiarsa JL Spontaneous synaptic activity is required for the formation of functional GABAergic synapses in the developing rat hippocampus J Physiol 2004 559 129 139 10.1113/jphysiol.2004.065060 15218067 PMC1665059

[B61] Liu Y Zhang LI Tao HW Heterosynaptic scaling of developing GABAergic synapses: dependence on glutamatergic input and developmental stage J Neurosci 2007 27 5301 5312 10.1523/JNEUROSCI.0376-07.2007 17507553 PMC3232185

[B62] Akerman CJ Cline HT Depolarizing GABAergic conductances regulate the balance of excitation to inhibition in the developing retinotectal circuit *in vivo* J Neurosci 2006 26 5117 5130 10.1523/JNEUROSCI.0319-06.2006 16687503 PMC6674233

[B63] Hartman KN Pal SK Burrone J Murthy VN Activity-dependent regulation of inhibitory synaptic transmission in hippocampal neurons Nat Neurosci 2006 9 642 649 10.1038/nn1677 16582905

[B64] Kerschensteiner D Morgan JL Parker ED Lewis RM Wong RO Neurotransmission selectively regulates synapse formation in parallel circuits *in vivo* Nature 2009 460 1016 1020 10.1038/nature08236 19693082 PMC2746695

[B65] Wong RO Collin SP Dendritic maturation of displaced putative cholinergic amacrine cells in the rabbit retina J Comp Neurol 1989 287 164 178 10.1002/cne.902870203 2477402

[B66] Cherry TJ Trimarchi JM Stadler MB Cepko CL Development and diversification of retinal amacrine interneurons at single cell resolution Proc Natl Acad Sci USA 2009 106 9495 9500 10.1073/pnas.0903264106 19470466 PMC2686638

[B67] Voinescu PE Kay JN Sanes JR Birthdays of retinal amacrine cell subtypes are systematically related to their molecular identity and soma position J Comp Neurol 2009 517 737 750 10.1002/cne.22200 19827163 PMC2814066

[B68] Feng G Mellor RH Bernstein M Keller-Peck C Nguyen QT Wallace M Nerbonne JM Lichtman JW Sanes JR Imaging neuronal subsets in transgenic mice expressing multiple spectral variants of GFP Neuron 2000 28 41 51 10.1016/S0896-6273(00)00084-2 11086982

